# Respiratory viral infections before the COVID-19 in Portugal: A single center study

**DOI:** 10.1016/j.heliyon.2024.e30894

**Published:** 2024-05-09

**Authors:** Vera Durão, Vera Clérigo, Paulo Durão, Ana Alfaiate, David Noivo, Fernando Durão, Maria Peres

**Affiliations:** a Hospital de Vila Franca de Xira, Lisboa, Portugal; b Hospital de São Bernardo, Setúbal, Portugal; c Instituto de Tecnologia Química e Biológica António Xavier, Universidade NOVA de Lisboa, Oeiras, Portugal; d Instituto Gulbenkian de Ciência, Oeiras, Portugal; e DECivil/CERENA, Técnico Lisboa, Universidade de Lisboa, Portugal

**Keywords:** Viral respiratory infections, Viral co-infection, Clinical features, Epidemiology, Before COVID-19

## Abstract

**Objectives:**

We aimed to describe the respiratory viruses (RV) found in respiratory samples from patients admitted to Hospital de São Bernardo, Setúbal, Portugal, between October 2019 and March 2020, and to correlate these with clinical features.

**Design:**

This retrospective study explored 948 fresh frozen naso/oropharyngeal swabs, tested by reverse transcription-polymerase chain reaction.

**Results:**

Rhinovirus/enterovirus, influenza, and respiratory syncytial virus (hRSV) were the most prevalent RV. Half of the patients fulfilled the acute respiratory infection (ARI) and/or influenza-like illness (ILI) criteria, with increasing age significantly reducing the risk of ARI and/or ILI. Hospital admission was more frequently observed in symptomatic patients, but the length of stay and mortality were significantly lower. Most (96.5 %) patients had a main respiratory diagnosis. In adults, the most prevalent was pneumonia, which particularly affected older patients, while in children, the most common was bronchiolitis. The number of hospital admissions was high. Sudden onset, shortness of breath, older age, and hRSV detection significantly increased the risk of hospital admission overall. In bronchiolitis, female gender significantly increased the risk of hospital admission, while older age significantly reduced this risk. Twenty patients died within the first month of sampling, and all were older adults. Older age and male gender significantly increased the risk of death.

**Conclusions:**

Respiratory viral infections can have a significant clinical impact, particularly in young infants with bronchiolitis and older adults with pneumonia. This study provides the first snapshot of the respiratory viral infections just before the outbreak of SARS-CoV-2 in Portugal, providing relevant clinical insights.

## 1 Introduction

Acute respiratory infections (ARI) are prevalent worldwide and a leading cause of death in developing countries [1,2]. Viral infections are the main cause of ARI [3], imposing a significant economic burden due to lost productivity and healthcare utilization [4]. The common cold is the most frequently encountered infectious syndrome in humans, and influenza has been a major cause of mortality and serious morbidity worldwide [3]. While trends in influenza and, more recently, SARS-CoV-2 infections are closely monitored, other respiratory pathogens are given less attention [4]. Advancements in molecular diagnostic techniques have led to a growing recognition of the role of respiratory viruses (RV) in diverse respiratory infections [3], and reverse transcription-polymerase chain reaction (rt-PCR) tests that simultaneously detect multiple respiratory pathogens have become the diagnostic modality of choice in most laboratories. However, careful clinical interpretation of laboratory data is imperative, since the detection of viral genome may not correlate with viral replication, possibly reflecting latency or prolonged shedding unrelated to the current symptoms [2].

In Portugal, the National Influenza Surveillance Program (NISP) ensures influenza epidemiological surveillance by reporting and testing Influenza-like illness (ILI) cases [5]. Testing for other RV is performed in cases of ILI with a negative test for influenza. Hence, influenza co-infections are not included in the scope of the program, nor are patients with non-influenza viral detection without ILI.

This study describes all the RV identified in the respiratory samples sent for virology testing in the months preceding the first official case of SARS-CoV-2 infection in Portugal and characterizes their clinical impact. The absence of potentially undetected early cases of SARS-CoV-2 was confirmed by testing a selected subpopulation.

## 2 Methods

### 2.1 Sampling and patient inclusion criteria

Upper airway samples were collected and tested between October 1st, 2019, and March 15th, 2020, at *Hospital de São Bernardo* (HSB), Setúbal. These samples, stored, fresh frozen, at the Immunology and Molecular Biology (IMB) laboratory, consisted of naso/oropharyngeal swabs from patients admitted to the emergency department of HSB, or inpatients, who had undergone respiratory pathogen testing by rt-PCR (BioFire® FilmArray® Respiratory Panel (RP2), Biomeriéux). The panel tested for influenza (A(H1) pdm09, A(H3) and B), rhinovirus/enterovirus (hRV/EnV), respiratory syncytial virus (hRSV), coronaviruses (hCoV) (HKU1, OC43, NL63, 229E), adenovirus (AdV), metapneumovirus (hMPV) and parainfluenza viruses (PIV) (PIV-1, PIV-2, PIV-3, and PIV-4). Non-identifying patient clinical data was collected retrospectively using the hospital intranet software, *SClinic*. The clinical features analyzed included 1) demographics, 2) month of sampling/testing, 3) number of hospital, Intermediate Care Unit and Intensive Care Unit (ICU) admissions at the time of sampling, 4) length of stay, 5) main respiratory diagnoses, 6) symptoms included in the standard European Union (EU) definition of ARI (sudden onset of symptoms (≤3 days), and at least one of four respiratory symptoms: cough, sore throat, shortness of breath or coryza) and ILI cases (sudden onset of symptoms, at least one of four systemic symptoms: fever, malaise, headache and myalgia, and at least one of three respiratory symptoms: cough, sore throat and shortness of breath) [6], and 7) death from all causes within the first four weeks following respiratory sampling. Clinician's judgement that the illness was due to an infection included in the EU ARI case definition was deemed present in all cases. Patients admitted to the hospital for treatment as inpatients (n = 371) or already inwards by the time the sample was taken (n = 83) were considered inpatients. The patients already inwards were all adults.

To rule out early, undetected cases of SARS-Cov-2 in Portugal, HSB and *Instituto Gulbenkian de Ciência* (IGC) teamed up to perform certified RNA COVID-19 tests on a subset of samples. Written informed consents were obtained from patients included in SARS-CoV-2 screening. Samples from adult (≥18y.o.) inpatients with the main diagnoses of respiratory infection or fever of unknown origin were included. In 2021, 124 swabs in Amies medium were inactivated at the IMB Lab and transported to the IGC in a cooler with freezer bags. Most samples (66.1 %) were originally collected from December 2019 to February 2020. Seventy (56.9 %) samples were from women, and the mean age was 71.7 yr. In IGC, the samples were submitted to a standard procedure certified by INSA and tested for SARS-CoV-2 by rt-PCR. To confirm sample viability, the amplification of the human gene coding for the RNA polymerase was used as a control. One-hundred and twenty-three samples amplified the control gene and were further assessed. All tested negative for SARS-CoV-2.

### 2.2 Statistics

Symptoms, ILI, ARI, RV, co-infection, death, and hospital admission were coded as binary variables. To evaluate associations between independent and dependent variables and to compute probabilities of events, the binary logistic regression modeling approach was selected, considering the dichotomous nature of the dependent variables. Independent variables analyzed for the dependent variables “hospital admission” and “death” included: gender, age, symptoms (sudden onset, fever, malaise, myalgia, headache, cough, shortness of breath, sore throat and coryza), detected RV (hRV/EnV, RSV, influenza A, influenza B, AdV, hMPV, coronaviruses OC43, NL63, 229E and HKU1, and parainfluenza viruses 1, 2, 3 and 4), co-infection, and ARI or ILI criteria. To avoid cofounding results, ARI and ILI were additionally incorporated as independent variables, excluding independent individual symptoms, since ARI and ILI result from the combination of these symptoms. Independent variables for the dependent variable “ARI and/or ILI” included: age, gender, RV, co-infection, and hospital admission. Age, gender, individual symptoms, ARI, and ILI were also tested as independent variables to predict the risk of specific viral detection. Selection of significant independent variables (with predictive power) was done by using the Forward-Stepwise (Likelihood-Ratio) Method and the adjustment of the coefficients of the retained independent variables of the logistic function was done by maximizing the log-likelihood function using a nonlinear optimization algorithm. Additional options included the model goodness of fit tests (Omnibus Tests of Model Coefficients, The Cox and Snell pseudo-R2 and the Nagelkerke pseudo-R2 and Hosmer-Lemeshow Test) and the classification tables to evaluate the overall predictive accuracy for a probability cut value determined from the ROC curve with coordinates corresponding approximately to equal values of sensitivity and specificity. Logistic regression was also performed for subgroups of patients with bronchiolitis and pneumonia. A bivariate analysis was performed to compare the subgroups of patients with and without ARI/ILI criteria using the chi-square test and the *t*-test. To compare viral detection between children/adolescents and adults the two-proportion z-test was used. Descriptive statistics were used for the basic analysis of the data. All analyses were conducted using the software EXCEL (Microsoft 365) and IBM SPSS Statistics 28.

## 3 Results

Nine hundred and forty-eight naso/oropharyngeal swabs were screened, and RV were identified in 630 (66.4 %). The prevalence of samples with viral detection was significantly higher among children/adolescents than adults (92.6 % and 52.9 %, respectively, z-score = 12.27, p < 0.00001). Of the samples with viral identification, 330 (52.4 %) were from adults, predominantly women (n = 199, 60.3 %). Of the samples from children/adolescents, 215 (71.7 %) were from children ≤2 yr, with a slight male predominance (n = 174, 58.0 %).

Most samples with viral detection came from inpatients (n = 454, 72.1 %), including 248 (75.1 %) adults and 163 (54.3 %) children ≤2 yr. Fifteen (3.3 %) were or had been admitted to ICUs, and ten (2.2 %) to Intermediate Care Units. These were all adults. The mean hospital length of stay for children/adolescents was 4.28 ± 3.94 days, while for adults it was 11.5 ± 12.2 days.

### 3.1 Respiratory viruses detected between October 2019 and March 2020

In the 630 samples included it was possible to identify 823 RV. A quarter (n = 164, 26.0 %) of the samples detected at least two different RV simultaneously (including 32 samples with the detection of three RV). Most (n = 115, 70.1 %) multiple viral infections (MVI) were found in children ≤5 yr, particularly when considering the detection of three RV (n = 28/32, 87.5 %). Overall, hRV/EnV was the respiratory virus most frequently reported (n = 250, 39.7 %), followed by influenza (n = 164, 26.0 %), and hRSV (n = 151, 24.0 %). Other less frequent RV included, in order of frequency: AdV, hMPV, hCoV and PIV (Table 1). In adults, the most common were hRV/EnV (37.1 %) and influenza (25.9 %), while in children/adolescents, the most prevalent were hRV/Env (25.0 %) and hRSV (23.0 %). Samples with MVI identified a total of 357 RV. The RV most frequently found in these samples were: hRV/EnV (n = 94, 26.3 %), AdV (n = 72, 20.2 %), hRSV (n = 62, 17.4 %) and influenza (n = 40, 11.2 %) (Supplementary Tables I and II). Influenza co-infections most frequently involved influenza A (n = 27, 67.5 %), and the most common were with AdV (n = 16, 40.0 %) and hRV/Env (n = 11, 27.5 %).

**Table 1:** **Baseline characteristics of the samples with viral detection.** ARI, acute respiratory infection; ILI, influenza-like illness; MVI, multiple viral infection; hRV/EnV, human rhinovirus/enterovirus; hRSV, respiratory syncytial virus; Infl. A, influenza A; AdV, adenovirus; hMPV, human metapneumovirus; Infl. B, influenza B; hCoV, human coronaviruses; PIV, parainfluenza viruses. n and (%) stand for number of cases and the corresponding percentage, respectively.

Samples with viral detection:	n (%)
October 2019	27 (4.3)
November 2019	53 (8.4)
December 2019	118 (18.7)
January 2020	183 (29.0)
February 2020	169 (26.8)
March 2020	80 (12.7)
Adults	330 (52.4)
Children/adolescents	300 (47.6)
ARI and/or ILI	348 (55.2)
Hospital admission	454 (72.1)
Death (all causes) in the 1st month	20 (3.2)
Samples with MVI	164 (26.0)
Total of samples with viral detection	**630 (100**)
Respiratory virus:	**n (%)**
hRV/EnV	250 (30.4)
hRSV	151 (18.4)
Influenza A	111 (13.5)
AdV	81 (9.8)
hMPV	76 (9.2)
Influenza B	53 (6.4)
hCoV HKU1	39 (4.7)
hCoV NL63	14 (1.7)
PIV-1	14 (1.7)
hCoV OC43	11 (1.3)
PIV-3	7 (0.8)
PIV-4	7 (0.8)
hCoV 229E	6 (0.7)
PIV-2	3 (0.4)
Total of respiratory viruses	**823 (100**)

**Fig. 1:**
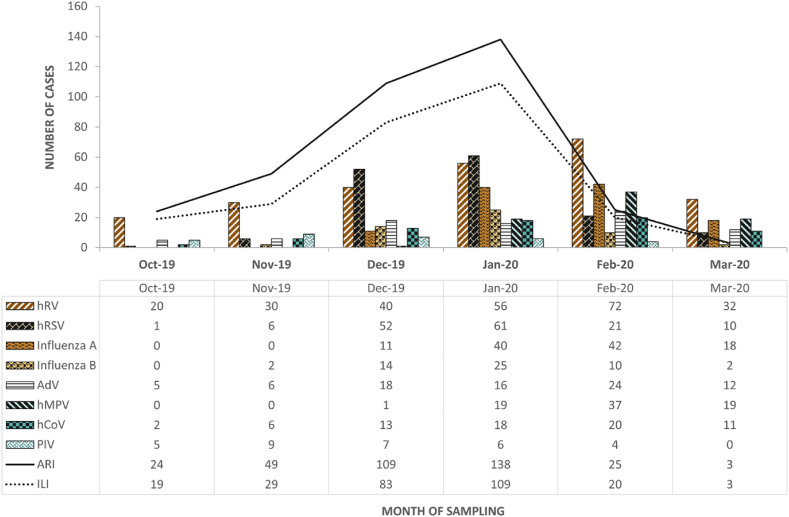
**Number of respiratory viruses, Influenza-like illness (ILI), and Acute Respiratory Infection (ARI) cases, determined by the month of sampling**. hRV, human rhinovirus/enterovirus; hRSV, human respiratory syncytial virus; AdV, adenovirus; hMPV, human metapneumovirus; hCoV, human coronaviruses; PIV, parainfluenza viruses; ARI, acute respiratory infection; ILI, Influenza-like illness; Oct-19, October 2019; Nov-19, November 2019; Dec-19, December 2019; Jan-20, January 2020; Feb-20, February 2020; Mar-20, March 2020.

Most RV were detected in January 2020 (38.25 %), February 2020 (36.51 %), and December 2019 (24.76 %) (Fig. 1).

### 3.2 Symptoms, ILI, and ARI criteria

Of the 630 samples with viral detection, 348 (55.2 %) were from patients with ARI criteria and 263 (41.7 %) from patients with ILI. The number of ILI and ARI cases, according to the month of sampling, is illustrated in Fig. 1. The comparison of clinical features and detected RV in the subgroups of patients with and without ILI or ARI criteria is depicted in Table 2.

**Table 2:** **- Clinical features and detected respiratory viruses in subgroups with and without (W/o) ILI or ARI criteria.** hRV/EnV, human rhinovirus/enterovirus; hRSV, human respiratory syncytial virus; AdV, adenovirus; hMPV, human metapneumovirus; hCoV, human coronaviruses; PIV, parainfluenza viruses; ARI, Acute Respiratory Infection; ILI, Influenza-like illness. n and (%) stand for number of cases and the corresponding percentage, respectively.

Total	W/o ARI/ILI, n (%)	ARI/ILI, n (%)		
	282 (100)	348 (100)	**χ**^**2**^**observed**	**p-value**
**<=2 y.o.**	71 (25.2)	144 (41.4)	18.1895	0.0000
**>=18 < 65 y.o.**	72 (25.5)	74 (21.3)	1.5934	0.2068
**>=65 y.o.**	101 (35.8)	83 (23.8)	10.7856	0.0010
**Male gender**	136 (48.2)	169 (48.6)	0.0071	0.9331
**Month of viral detection**				
**October 2019**	3 (1.1)	24 (6.9)	12.9190	0.0003
**November 2019**	4 (1.4)	49 (14.1)	32.4133	0.0000
**December 2019**	9 (3.2)	109 (31.3)	80.9780	0.0000
**January 2020**	45 (16.0)	138 (39.7)	42.4447	0.0000
**February 2020**	144 (51.1)	25 (7.2)	152.7963	0.0000
**March 2020**	77 (27.3)	3 (0.9)	98.2504	0.0000
**Respiratory Viruses**				
**hRV/EnV**	112 (39.7)	138 (39.7)	0.0002	0.9876
**Influenza**	88 (31.2)	76 (21.8)	7.0975	0.0077
**hRSV**	39 (13.8)	112 (32.2)	28.7954	0.0000
**AdV**	40 (14.2)	41 (11.8)	0.8027	0.3703
**hMPV**	55 (19.5)	21 (6.0)	26.6391	0.0000
**hCoV**	33 (11.7)	37 (10.6)	0.1806	0.6709
**PIV**	7 (2.5)	24 (6.9)	6.4878	0.0109
**Viral Co-infection**	75 (26.6)	89 (25.6)	0.0843	0.7715
**Diagnosis**				
**Pneumonia**	93 (33.0)	123 (35.3)	0.3871	0.5338
**ATB**	124 (44.0)	108 (31.0)	11.2066	0.0008
**Bronchiolitis**	27 (9.6)	89 (25.6)	26.5461	0.0000
**Other Resp. Inf.**	16 (5.7)	26 (7.5)	0.8089	0.3685
**Other Diagnoses**	20 (7.3)	2 (0.6)	19.6337	0.0000
**Outcomes**				
**Hospital admission**	182 (64.5)	272 (78.2)	14.3574	0.0002
**Mortality (4 weeks)**	13 (4.6)	7 (2.0)	3.4216	0.0643
			t observed	p-value
**Age (years), mean ± SD**	42.3 ± 34.6	30.0 ± 34.1	4.4724	0.0000
**Hospital length of stay (days)**	9.45 ± 9.9	7.6 ± 9.9	2.3323	0.02

There was no significant association between ILI or ARI and the detection of influenza or other RV (p = 0.516 and 0.884, respectively; see Fig. 3 for the odds ratio of significant independent clinical features, and Supplementary Table III for the individual clinical features according to the detected respiratory virus).

### 3.3 Respiratory diagnoses

**Fig. 2:**
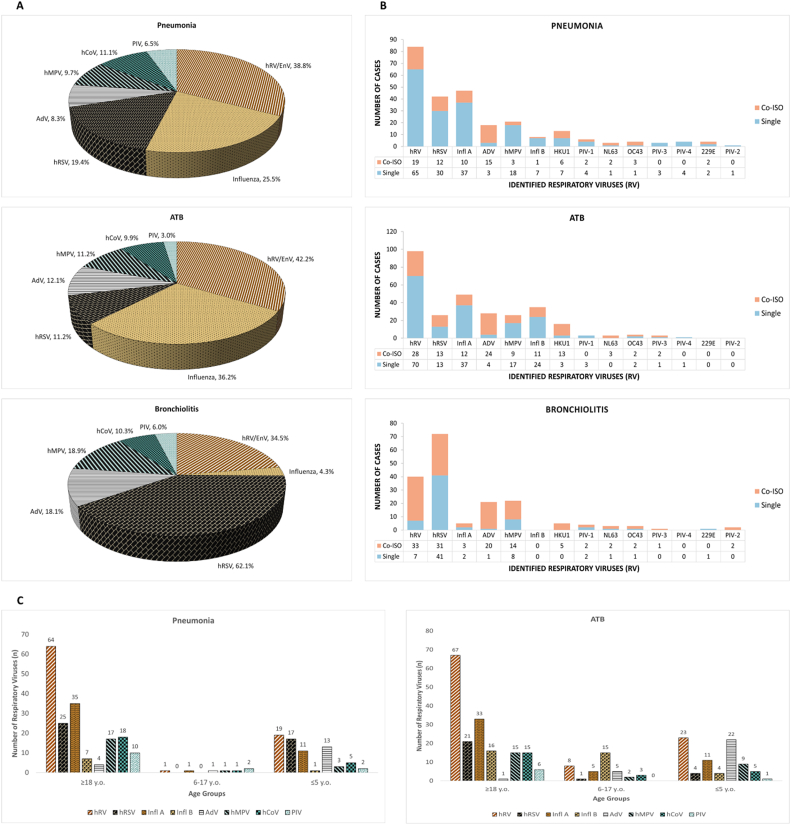
**(A) Viral etiology of pneumonia, acute tracheobronchitis (ATB), and bronchiolitis**, **including (B) cases of co-infection**. **(C) Viral etiology of pneumonia and acute tracheobronchitis (ATB), according to age group**. Co–ISO, co-infection; Single, identification of a single respiratory virus; hRV, human rhinovirus; hRSV, human respiratory syncytial virus; Infl. A, influenza A; AdV, adenovirus; hMPV, human metapneumovirus; Infl. B, influenza B; hCoV, human coronaviruses; PIV, parainfluenza viruses.

Most patients (n = 608, 96.5 %) had a main respiratory diagnosis, including 328 (99.4 %) adults and 280 (93.3 %) children/adolescents. The most common were tracheobronchitis (n = 232, 38.2 %), pneumonia (n = 216, 35.5 %), and bronchiolitis (n = 116, 19.1 %). Two samples came from patients with unspecified main diagnoses. The RV, clinical characteristics, and outcomes, as determined by the main respiratory diagnosis, are presented in Table 3. The viral etiology of pneumonia, tracheobronchitis, and bronchiolitis, including cases of co-infection, and the viral etiology of pneumonia and tracheobronchitis according to age group are depicted in Fig. 2.

**Table 3:** **Respiratory viruses, clinical characteristics, and outcomes, as determined by the main respiratory diagnosis**. Samples from inpatients with pneumonia were mostly (n = 150, 75.76 %) taken in the first 48 h of admission. ORI, other respiratory infections included: acute nasopharyngitis, recurrent wheezing, tonsillitis, laryngitis, and unspecified respiratory infection. Other Dx, other diagnoses included: fever of unknown origin, myositis, myopericarditis, exanthema, unspecified headache, encephalitis, febrile neutropenia, acute otitis media, sepsis of unknown focus, pulmonary edema, and unspecified pleural effusion. hRV EnV, human rhinovirus/enterovirus; hRSV, human respiratory syncytial virus; AdV, adenovirus; hMPV, human metapneumovirus; hCoV, human coronaviruses; PIV, parainfluenza viruses; Viral Co-inf., viral co-infection. n and (%) stand for number of cases and the corresponding percentage, respectively.

	Pneumonia n (%)	ATB n (%)	BRL n (%)	ORI n (%)	Other Dx n (%)
**Age (years), mean ± SD**	54.3 ± 32.6	41.6 ± 31.8	0.4 ± 0.8	12.9 ± 24.7	18.1 ± 30.6
**≤2 y.o**.	35 (16.2)	32 (13.8)	114 (98.3)	25 (59.5)	9 (40.9)
**≥ 65 y.o**.	109 (50.5)	68 (29.3)	0 (0.0)	3 (7.1)	4 (18.2)
**Male gender**	108 (50.0)	93 (40.1)	67 (57.8)	24 (57.1)	12 (54.5)
**hRV/EnV**	84 (38.8)	98 (42.2)	40 (34.5)	18 (42.9)	6 (27.3)
**Influenza**	55 (25.5)	84 (36.2)	5 (4.3)	7 (16.7)	9 (40.9)
**hRSV**	42 (19.4)	26 (11.2)	72 (62.1)	7 (16.7)	4 (18.2)
**AdV**	18 (8.3)	28 (12.1)	21 (18.1)	12 (28.6)	1 (4.5)
**hMPV**	21 (9.7)	26 (11.2)	22 (18.9)	4 (9.5)	3 (13.6)
**hCoV**	24 (11.1)	23 (9.9)	12 (10.3)	6 (14.3)	5 (22.7)
**PIV**	14 (6.5)	7 (3.0)	7 (6.0)	3 (7.1)	0 (0–0)
**Viral Co-inf**.	33 (15.3)	57 (24.6)	52 (44.8)	16 (38.1)	5 (22.7)
**Hospital Admission**	198 (91.7)	117 (50.4)	100 (86.2)	26 (61.9)	13 (59.1)
**Mortality (4 weeks)**	15 (6.9)	5 (2.2)	0 (0.0)	0 (0.0)	0 (0.0)
**Total**	216 (100)	232 (100)	116 (100)	42 (100)	22 (100)

**Fig. 3:**
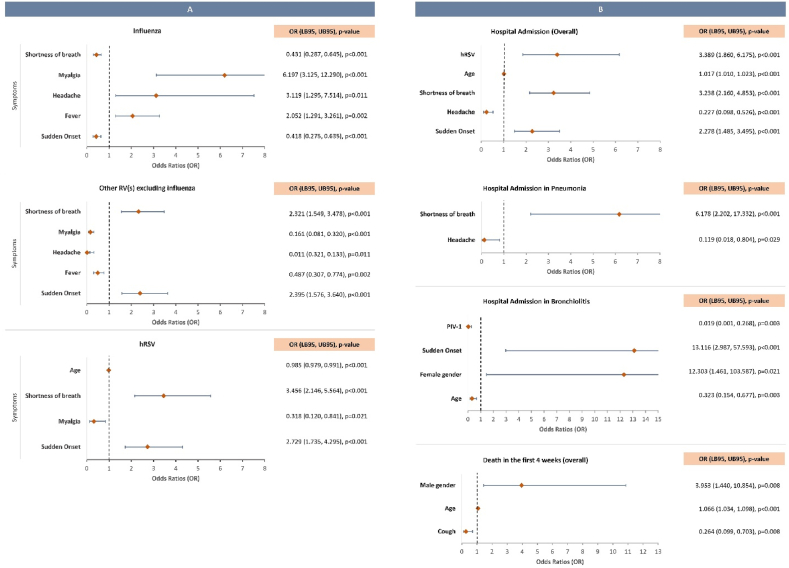
**Forest plots of the odds ratios (OR) and 95 % confidence intervals (CI) for: (A) the risk of detecting influenza, other respiratory viruses (RV) excluding influenza, and hRSV (human respiratory syncytial virus)** – Myalgia, headache and fever significantly increased the risk of influenza detection while sudden onset and shortness of breath decreased this risk. On the other hand, sudden onset and shortness of breath significantly increased the risk of detecting other RV excluding influenza while myalgia, headache and fever decreased this risk. This positive association was accentuated when analyzing the risk of hRSV detection exclusively. Myalgia and older age presented a negative association with hRSV positivity; **(B) the risk of hospital admission (overall and in subgroups with pneumonia and bronchiolitis) and death within the first 4 weeks following respiratory sampling (overall)** – Sudden onset, shortness of breath, older age, and hRSV detection significantly increased the overall risk of hospital admission; headache decreased the risk of this event. In patients with pneumonia, shortness of breath significantly increased the risk of hospital admission, while headache significantly reduced this risk. In patients with bronchiolitis, sudden onset and female gender significantly increased the risk of hospital admission; older age and PIV-1 detection significantly decreased this risk. Older age and male gender significantly increased the risk of death while cough significantly reduced the risk of this outcome within the first month. X-axis represents the odds ratios (OR) and 95 % confidence intervals (CI) in the following order: OR [Lower Bound 95, Upper Bound 95], p-value. RV(s), respiratory viruses; hRSV, human respiratory syncytial virus; PIV-1, parainfluenza virus 1.

### 3.4 Risk of hospital admission and death

Sudden onset, shortness of breath, older age, and hRSV detection significantly increased the risk of hospital admission overall (Fig. 3). After adjusting for other variables, excluding individual symptoms, ARI significantly increased the risk of hospital admission (OR 2.295 [1.535–3.432], p-value <0.001). In patients with bronchiolitis, female gender significantly increased the risk of hospital admission, whereas older age significantly reduced this risk.

Twenty patients (3.2 %) died within the first four weeks following respiratory sampling (Table 1). The mean age of this subgroup was 77.8 ± 9.8 yr, and most were men (n = 13, 65.0 %). There were no fatality cases in children/adolescents. Of the twenty deceased patients, fifteen had pneumonia. Older age and male gender significantly increased the risk of death (Fig. 3). After adjustment for other variables, including or excluding individual symptoms, ILI and co-infection were not significantly associated with the risk of hospital admission (p = 0.280 and 0.788, respectively) or death (p = 0.167 and 0.117).

## 4 Discussion

This study analyzed naso/oropharyngeal swabs sent for rt-PCR viral testing between October 2019 and March 2020. Viral detection rate was higher in the pediatric population, likely due to a higher viral load, compared to adults [7]. hRV/EnV was the respiratory virus most frequently identified, aligning with previous reports from Northern Taiwan [8] and Parma (Italy) [9] for the 2019/2020 season. Among adults, hRV/EnV and influenza were the predominant viruses, and in children, hRV/EnV and hRSV were the most common, consistent with previous findings [5,8,9]. Influenza and hRSV displayed expected pre-pandemic monthly patterns [5,9,10], peaking in January. Interestingly, hRV/EnV, which has been described as having peak rates in the spring and autumn and a steady distribution in the winter months with heightened disease severity [10], presented an increasing incidence from October 2019 to February 2020, reaching its peak.

Systemic symptoms such as fever, headache, and myalgia significantly increased the risk of influenza detection, while shortness of breath significantly decreased this risk, in agreement with the latest 2018/2019 Portuguese NISP report. On the other hand, shortness of breath significantly increased the risk of hRSV detection, while myalgia was negatively associated to hRSV-positivity, in accordance with the findings by Sáez-López et al. [11] regarding surveillance case definitions for hRSV infection in Portugal from 2010 to 2018.

Half of the patients who underwent viral testing exhibited symptoms indicative of ARI and/or ILI. Increasing age significantly reduced the risk of ARI and/or ILI (OR 0.987 [0.980–0.994], p-value <0.001), and the monthly distribution of ARI and/or ILI cases mirrored the monthly distribution of hRSV, and influenza. Hospital admission more frequently occurred in symptomatic patients. However, the length of stay and mortality tended to be significantly lower, likely reflecting the younger demographic of ARI and/or ILI patients (including a significantly higher proportion of children with bronchiolitis). Regardless of ARI and/or ILI criteria, most (96.5 %) patients had a main respiratory diagnosis. In adults, pneumonia (n = 164, 50.0 %) was the most prevalent, particularly affecting patients ≥65 yr (50.5 %). Although RV frequencies slightly differed between adults and children with pneumonia, since AdV and hRSV played important roles as etiological agents in children ≤5 yr, hRV/EnV remained the most prevalent pathogen in both age groups.

Two large epidemiological studies [12,13] showed that viral agents were the most common pathogens in community-acquired pneumonia requiring hospitalization: hRV/EnV and influenza were the RV most frequently found in adults and, consistent with our results, hRSV and hRV/EnV were the most prevalent in children.

A pathological role for hRV/EnV in severe lower respiratory-tract infections has been described in infants and adults with co-morbidities [14]. Although its clinical interpretation may be challenging, in this study, the majority (n = 76, 90.5 %) of patients with community-acquired pneumonia [samples collected in the first 48 h of admission] and hRV/EnV detection (n = 84) were admitted to the hospital and treated as inpatients. Also, hRV/EnV was detected in the respiratory samples of 55 % of the deceased patients.

Acute viral bronchiolitis, a common clinical syndrome affecting infants and young children, and one of the most substantial health burdens at this age, was primarily attributed to hRSV, in agreement with previous reports [15,16]. This condition is typically described in children until the age of 12–24 months. However, in this study, two patients aged 3 and 5 yr were diagnosed with bronchiolitis. Both patients met the clinical criteria for a first episode of bronchiolitis, and viral agents were detected: hRSV in one and viral co-infection (AdV, CoV-HKU1 and hMPV) in the other. A large percentage of MVI was detected (∼45 %), and hRV/EnV, which has also been described as an important cause of bronchiolitis [16,17], was the agent most frequently involved. AdV and hMPV [16,17], which have also been implicated as etiological agents, were mostly detected in the setting of co-infection.

Co-infection rates were observed in a quarter of all samples, but in 45.4 % of children ≤5 yr, making them notably higher in young children [5,7]. These rates were higher than those described for the 2018/2019 Portuguese winter season (14 % in children ≤4 yr) [5] and the 2019/2020 season in Parma (overall MVI rate of 19.8 %) [9]. The impact of MVI on severe viral respiratory disease remains uncertain [[18], [19], [20], [21]]. Recent research even suggests that MVI may be protective against severe disease in some cases, unlike viral-bacterial infections, which most likely contribute to worse outcomes [[20], [21], [22]]. In this study, viral co-infection was not significantly associated to hospital admission or death. No cases of viral co-infection were detected in the patients admitted to the ICU or intermediate care units.

Acute viral infection can cause significant morbidity and mortality, particularly in the elderly [23], an observation corroborated by our results. However, in children with bronchiolitis, older age significantly reduced the risk of hospital admission, emphasizing the burden of respiratory viral infections in younger infants [15,16]. Our results suggest that men with viral pneumonia experience worse outcomes, consistent with the literature [24]. Yet, in young children with bronchiolitis, female gender significantly increased the risk of hospital admission. This stark contradiction with the literature [15] warrants further investigation into the immunomodulatory effects of gender in infants with acute viral bronchiolitis.

Shortness of breath is often accompanied by an increased respiratory rate, a well-known severity assessment parameter for community-acquired pneumonia [25] and acute bronchiolitis [26], which may explain why this symptom was found to be an independent risk factor for hospital admission. A rapidly evolving disease that prompts a hospital visit within the first three days of onset may reflect a more aggressive and severe disease, explaining why sudden onset also significantly increased this risk. hRSV detection significantly increased the risk of hospital admission, corroborating previous findings [[27], [28], [29], [30]] of a significant health burden for this viral infection, particularly in young children.

This retrospective analysis included a significant number of patients and was based on the results of the rt-PCR test, the most used method for viral detection. Retrospective nature might have limited correct identification of ARI and ILI cases. Nonetheless, the included symptoms most likely reflect the clinician's judgement regarding the severity of an emergency episode. Bacterial superimposed infection and co-morbidities, usually described as risk factors for severe disease, especially in the elderly, were not analyzed, and this should be further investigated in a properly dedicated study. Notably, this study analyzed the pre-COVID-19 pandemic period, offering clinical insights into respiratory viral infections just before the outbreak of SARS-CoV-2 in Portugal.

## 5 Conclusions

Viral respiratory infections can have a significant clinical impact, especially in young children with bronchiolitis, who tend to be more symptomatic and have an increased risk of hospital admission, and older male adults, who present an increased risk of hospital admission and death, particularly due to pneumonia. The clinical meaning of viral detection due to latency or prolonged shedding remains unclear since no viral replication or viral infectivity tests were performed. Nevertheless, viral detection in this setting might signal the trigger of a series of events, in susceptible and unfit patients with chronic diseases, that culminate in the development of late complications with significant morbidity. Further investigation is required to develop and implement laboratory techniques capable of detecting active viral infection, and to determine the clinical features associated with a higher likelihood of severe disease in respiratory viral infections.

## Ethics statement

This study was approved by the Ethical Committee of *Centro Hospitalar de Setúbal* through the Research and Development Office (GID – *Gabinete de Investigação e Desenvolvimento*) on July 8th, 2020, with the approval reference number: 020_2020F. All participants/patients (or their proxies/legal guardians) provided written informed consent to participate in the SARS-CoV-2 screening.

## Funding source

This work was supported by project 467, “Identification and genetic sequencing of early COVID-19 positive cases in Portugal.“, *Fundação para a Ciência e Tecnologia* (10.13039/501100001871FCT).

## Data availability statement

Data included in the article is given either in supplemental material or as referenced in the article.

## CRediT authorship contribution statement

**Vera Durão:** Writing – review & editing, Writing – original draft, Visualization, Validation, Supervision, Software, Resources, Project administration, Methodology, Investigation, Funding acquisition, Formal analysis, Data curation, Conceptualization. **Vera Clérigo:** Writing – review & editing, Visualization, Validation, Supervision, Project administration, Investigation, Formal analysis, Data curation. **Paulo Durão:** Writing – review & editing, Visualization, Validation, Supervision, Resources, Project administration, Methodology, Investigation, Funding acquisition, Formal analysis, Conceptualization. **Ana Alfaiate:** Writing – review & editing, Visualization, Project administration, Investigation, Data curation. **David Noivo:** Writing – review & editing, Visualization, Project administration, Investigation, Data curation. **Fernando Durão:** Visualization, Supervision, Software, Methodology, Investigation, Formal analysis. **Maria Peres:** Writing – review & editing, Visualization, Resources, Methodology, Investigation, Data curation.

## Declaration of competing interest

The authors declare that they have no known competing financial interests or personal relationships that could have appeared to influence the work reported in this paper.
