# Supplementary material for: Respiratory viral infections before the COVID-19 in Portugal: A single center study
Source: Heliyon. 2024 May 9;10(10):e30894. doi: 10.1016/j.heliyon.2024.e30894 (PMC11109810; doi:10.1016/j.heliyon.2024.e30894)
Supplement: Multimedia component 1 [file mmc1.docx]

**SUPPLEMENTARY MATERIAL**

**Table I - Frequencies of viral co-isolates (≥ 2 RV).** hRV, human rhinovirus/enterovirus; hRSV, human respiratory syncytial virus; Infl A, influenza A; AdV, adenovirus; hMPV, human metapneumovirus; Infl B, influenza B; HKU1, human coronavirus HKU1; PIV-1, parainfluenza virus 1; NL63, human coronavirus NL63; OC43, human coronavirus OC43; PIV-3, parainfluenza virus 3; PIV-4, parainfluenza virus 4; 229E, human coronavirus 229E; PIV-2, parainfluenza virus 2.

|  | **hRV** | **hRSV** | **Infl A** | **AdV** | **hMPV** | **Infl B** | **HKU1** | **PIV-1** | **NL63** | **OC43** | **PIV-3** | **PIV-4** | **229E** | **PIV-2** |
| --- | --- | --- | --- | --- | --- | --- | --- | --- | --- | --- | --- | --- | --- | --- |
| **hRV** |  | 33 | 9 | 32 | 18 | 2 | 7 | 3 | 0 | 4 | 1 | 0 | 2 | 1 |
| **hRSV** | 33 |  | 6 | 18 | 4 | 3 | 6 | 0 | 4 | 1 | 0 | 0 | 0 | 1 |
| **Infl A** | 9 | 6 |  | 13 | 1 | 0 | 2 | 1 | 2 | 0 | 2 | 0 | 0 | 0 |
| **AdV** | 32 | 18 | 13 |  | 13 | 3 | 9 | 2 | 4 | 1 | 2 | 1 | 0 | 1 |
| **hMPV** | 18 | 4 | 1 | 13 |  | 0 | 4 | 0 | 0 | 0 | 0 | 1 | 0 | 0 |
| **Infl B** | 2 | 3 | 0 | 3 | 0 |  | 5 | 0 | 0 | 0 | 0 | 0 | 0 | 0 |
| **HKU1** | 7 | 6 | 2 | 9 | 4 | 5 |  | 0 | 1 | 2 | 0 | 0 | 0 | 0 |
| **PIV-1** | 3 | 0 | 1 | 2 | 0 | 0 | 0 |  | 0 | 0 | 0 | 0 | 0 | 0 |
| **NL63** | 0 | 4 | 2 | 4 | 0 | 0 | 1 | 0 |  | 0 | 0 | 0 | 0 | 0 |
| **OC43** | 4 | 1 | 0 | 1 | 0 | 0 | 2 | 0 | 0 |  | 0 | 0 | 0 | 0 |
| **PIV-3** | 1 | 0 | 2 | 2 | 0 | 0 | 0 | 0 | 0 | 0 |  | 0 | 0 | 0 |
| **PIV-4** | 0 | 0 | 0 | 1 | 1 | 0 | 0 | 0 | 0 | 0 | 0 |  | 0 | 0 |
| **229E** | 2 | 0 | 0 | 0 | 0 | 0 | 0 | 0 | 0 | 0 | 0 | 0 |  | 0 |
| **PIV-2** | 1 | 1 | 0 | 1 | 0 | 0 | 0 | 0 | 0 | 0 | 0 | 0 | 0 |  |

**Table II - Description of the 32 cases of co-infection with three respiratory viruses**. hRV, human rhinovirus/enterovirus; hRSV, human respiratory syncytial virus; Infl A, influenza A; AdV, adenovirus; hMPV, human metapneumovirus; Infl B, influenza B; HKU1, human coronavirus HKU1; PIV-1, parainfluenza virus 1; NL63, human coronavirus NL63; OC43, human coronavirus OC43; PIV-3, parainfluenza virus 3; PIV-4, parainfluenza virus 4; 229E, human coronavirus 229E; PIV-2, parainfluenza virus 2.

|  | **hRV**  **+**  **hRSV** | **hRV**  **+**  **Infl. A** | **hRV**  **+**  **ADV** | **hRV**  **+**  **HKU1** | **hRSV**  **+**  **Infl. A** | **hRSV**  **+**  **AdV** | **AdV**  **+**  **Infl. A** | **AdV**  **+**  **hMPV** | **AdV**  **+**  **Infl. B** |
| --- | --- | --- | --- | --- | --- | --- | --- | --- | --- |
| **hRV** |  |  |  |  |  |  |  |  |  |
| **hRSV** |  |  |  |  |  |  |  |  |  |
| **Infl. A** | 1 |  |  |  |  |  |  |  |  |
| **AdV** | 5 | 2 |  |  | 1 |  |  |  |  |
| **hMPV** | 0 | 0 | 5 |  | 1 | 1 | 0 |  |  |
| **Infl. B** | 1 | 0 | 0 | 0 | 0 | 0 | 0 | 0 |  |
| **HKU1** | 0 | 0 | 3 | 0 | 0 | 2 | 0 | 1 | 1 |
| **PIV1** | 0 | 0 | 0 | 0 | 0 | 0 | 1 | 0 | 0 |
| **NL63** | 0 | 0 | 0 | 0 | 1 | 1 | 0 | 0 | 0 |
| **OC43** | 0 | 0 | 0 | 1 | 0 | 0 | 0 | 0 | 0 |
| **PIV3** | 0 | 0 | 0 | 0 | 0 | 0 | 2 | 0 | 0 |
| **PIV4** | 0 | 0 | 0 | 0 | 0 | 0 | 0 | 1 | 0 |
| **229E** | 0 | 0 | 0 | 0 | 0 | 0 | 0 | 0 | 0 |
| **PIV2** | 0 | 0 | 1 | 0 | 0 | 0 | 0 | 0 | 0 |
