# Supplementary material for: Respiratory viral infections before the COVID-19 in Portugal: A single center study
Source: Heliyon. 2024 May 9;10(10):e30894. doi: 10.1016/j.heliyon.2024.e30894 (PMC11109810; doi:10.1016/j.heliyon.2024.e30894)
Supplement: Multimedia component 2 [file mmc2.docx]

**SUPPLEMENTARY MATERIAL**

**Table III - Clinical features according to the detected respiratory virus.** hRV, human rhinovirus/enterovirus; hRSV, respiratory syncytial virus; Infl., influenza virus; AdV, adenovirus; hMPV, human metapneumovirus; HKU1, coronavirus HKU1; PIV1, parainfluenza virus 1; NL63, coronavirus NL63; OC43, coronavirus OC43; PIV3, parainfluenza virus 3; PIV4, parainfluenza virus 4; 229E, coronavirus 229E; PIV2, parainfluenza virus 2; Co-ISO, co-infection – refers to samples with the detection of two or more RV; ICU, intensive care unit; Intermediate CU, intermediate care unit; SdOnset, sudden onset; SoBreath, shortness of breath.

|  | **hRV** | **hRSV** | **Infl. A** | **AdV** | **hMPV** | **Infl. B** | **HKU1** | **PIV1** | **NL63** | **OC43** | **PIV3** | **PIV4** | **229E** | **PIV2** | **Total** |
| --- | --- | --- | --- | --- | --- | --- | --- | --- | --- | --- | --- | --- | --- | --- | --- |
| **Co-ISO, n (%)** | 94 (37.6) | 62 (41.1) | 27 (24.3) | 72 (88.9) | 32 (42.1) | 13 (24.5) | 28 (71.8) | 5  (35.7) | 9  (64.3) | 7  (63.6) | 3  (42.9) | 1  (14.3) | 2  (33.3) | 2  (66.7) | 357  (43.4) |
| **Age (years), mean±SD** | 38±36 | 23±35 | 39±31 | 5±13 | 34±37 | 26±26 | 36±36 | 31±35 | 21±33 | 40±32 | 58±40 | 36±33 | 65±33 | 5±9 | 36±35 |
| **Male gender, n (%)** | 129 (51.6) | 79 (52.3) | 62  (55.9) | 44 (54.3) | 35 (46.1) | 22 (41.5) | 18 (46.1) | 4  (28.6) | 7  (50.0) | 4  (36.4) | 2  (28.6) | 3  (42.9) | 4  (66.7) | 2  (66.7) | 415  (50.4) |
| **Symptoms** | | | | | | | | | | | | | | | |
| **SdOnset, n (%)** | 144 (57.6) | 117 (77.5) | 46 (41.4) | 41 (50.6) | 26 (34.2) | 36 (67.9) | 18 (46.2) | 12 (85.7) | 12 (85.7) | 8  (72.7) | 4  (57.1) | 5  (71.4) | 2  (33.3) | 3  (100.0) | 474  (57.6) |
| **Fever, n (%)** | 136 (54.4) | 97 (64.2) | 84 (75.7) | 53 (65.4) | 47 (61.8) | 46 (86.8) | 26 (66.7) | 6  (42.9) | 11 (78.6) | 7  (63.6) | 3  (42.9) | 4  (57.1) | 4  (66.7) | 2  (66.7) | 526  (63.9) |
| **Malaise, n (%)** | 58 (23.2) | 31 (20.5) | 23 (20.7) | 15 (18.5) | 28 (36.8) | 9  (17.0) | 7  (17.9) | 1  (7.1) | 4  (28.6) | 1  (9.1) | 0  (0.0) | 1  (14.3) | 1  (16.7) | 1  (33.3) | 180  (21.9) |
| **Headache, n (%)** | 6  (2.4) | 5  (3.3) | 6  (5.4) | 0  (0.0) | 4  (5.3) | 14 (26.4) | 0  (0.0) | 0  (0.0) | 1  (7.1) | 1  (9.1) | 0  (0.0) | 0  (0.0) | 0  (0.0) | 0  (0.0) | 37  (4.5) |
| **Myalgia, n (%)** | 14  (5.6) | 5  (3.3) | 16 (14.4) | 0  (0.0) | 3  (3.9) | 18 (34.0) | 1  (2.6) | 0  (0.0) | 0  (0.0) | 4  (36.4) | 0  (0.0) | 0  (0.0) | 0  (0.0) | 0  (0.0) | 61  (7.4) |
| **Cough, n (%)** | 186 (74.4) | 123 (81.5) | 81 (73.0) | 62 (76.5) | 55 (72.4) | 41 (77.4) | 28 (71.8) | 9  (64.3) | 10 (71.4) | 8  (72.7) | 5  (71.4) | 7  (100.0) | 4  (66.7) | 3  (100.0) | 622  (75.6) |
| **Sore throat, n (%)** | 31 (12.4) | 10  (6.6) | 19 (17.1) | 6  (7.4) | 11 (14.5) | 11 (20.7) | 1  (2.6) | 2  (14.3) | 1  (7.1) | 3  (27.3) | 0  (0.0) | 0  (0.0) | 0  (0.0) | 0  (0.0) | 95  (11.5) |
| **SoBreath, n (%)** | 164 (65.6) | 122 (80.8) | 60 (54.1) | 41 (50.6) | 54 (71.1) | 12 (22.6) | 21 (53.8) | 9  (64.3) | 7  (50.0) | 7  (63.6) | 4  (57.1) | 5  (71.4) | 5  (83.3) | 2  (66.7) | 513  (62.3) |
| **Coryza, n (%)** | 47 (18.8) | 49 (32.4) | 21 (18.9) | 27 (33.3) | 11 (14.5) | 14 (26.4) | 7  (17.9) | 5  (35.7) | 7  (50.0) | 1  (9.1) | 0  (0.0) | 0  (0.0) | 0  (0.0) | 1  (33.3) | 190  (23.1) |
| **Outcomes** | | | | | | | | | | | | | | | |
| **Hospital Admission, n (%)** | 184 (73.6) | 134 (88.7) | 72 (64.9) | 49 (60.5) | 57 (75.0) | 22 (41.5) | 27 (69.2) | 8 (57.1) | 10 (71.4) | 10  (90.9) | 6  (85.7) | 4  (57.1) | 5  (83.3) | 2  (66.7) | 590  (71.7) |
| **ICU, n (%)** | 8 (3.2) | 0 (0.0) | 4 (3.6) | 1 (1.2) | 0 (0.0) | 0 (0.0) | 1 (2.6) | 0 (0.0) | 0 (0.0) | 0 (0.0) | 0 (0.0) | 1(14.3) | 0 (0.0) | 0 (0.0) | 15(1.8) |
| **Intermediate CU** | 2 (0.8) | 2 (1.3) | 2 (1.8) | 0 (0.0) | 3 (3.9) | 0 (0.0) | 0 (0.0) | 1 (7.1) | 0 (0.0) | 0 (0.0) | 0 (0.0) | 0 (0.0) | 0 (0.0) | 0 (0.0) | 10(1.2) |
| **Deaths** (4 weeks) | 11 4.4) | 3 (2.0) | 3 (2.7) | 0 (0.0) | 0 (0.0) | 0 (0.0) | 3 (7.7) | 0 (0.0) | 0 (0.0) | 0 (0.0) | 0 (0.0) | 0 (0.0) | 1(16.7) | 0 (0.0) | 21(2.5) |
| **Total, n** | 250 | 151 | 111 | 81 | 76 | 53 | 39 | 14 | 14 | 11 | 7 | 7 | 6 | 3 | 823 |
